# Poly(lactic acid) Matrix Reinforced with Diatomaceous Earth

**DOI:** 10.3390/ma15186210

**Published:** 2022-09-07

**Authors:** Izabela Zglobicka, Magdalena Joka-Yildiz, Rafal Molak, Michal Kawalec, Adrian Dubicki, Jakub Wroblewski, Kamil Dydek, Anna Boczkowska, Krzysztof J. Kurzydlowski

**Affiliations:** 1 Faculty of Mechanical Engineering, Bialystok University of Technology, Wiejska 45C, 15-351 Bialystok, Poland; 2 Faculty of Civil Engineering and Environmental Sciences, Bialystok University of Technology, Wiejska 45E, 15-351 Bialystok, Poland; 3 Faculty of Materials Science and Engineering, Warsaw University of Technology, Woloska 141, 02-507 Warsaw, Poland

**Keywords:** PLA composites, diatomaceous earth, extrusion, biodegradable composites

## Abstract

The poly(lactic acid) (PLA) biodegradable polymer, as well as natural, siliceous reinforcement in the form of diatomaceous earth, fit perfectly into the circular economy trend. In this study, various kinds of commercial PLA have been reinforced with diatomaceous earth (DE) to prepare biodegradable composites via the extrusion process. The structure of the manufactured composites as well as adhesion between the matrix and the filler were investigated using scanning electron microscopy (SEM). Differential scanning calorimetry (DSC) analyses were carried out to determine crystallinity of PLA matrix as function of DE additions. Additionally, the effect of the ceramic-based reinforcement on the mechanical properties (Young’s modulus, elongation to failure, ultimate tensile strength) of PLA has been investigated. The results are discussed in terms of possible applications of PLA + DE composites.

## 1. Introduction

The latest trends in the development and manufacturing of novel materials are strictly related to the idea of the circular economy because of the environmental concerns as well as finite petroleum resources [1]. This approach particularly applies to polymer-based materials and results in increasing interest in research and development (R and D) work focused on biodegradable polymers.

One of the most promising and during the last decade the most intensively investigated biodegradable polymer is poly(lactic acid) (PLA). An advantage of the PLA is its commercial availability and remarkable properties which shall be tuned-up to meet the needs of specific applications. Properties of PLA can be further enhanced by various reinforcements: fibers (e.g., flax, kenaf, glass fibers) as well as particles (e.g., talc, hydroxyapatite, calcium carbonate) [2]. In the context of circular economy, diatomaceous earth—naturally occurring and consisting of fossilized remains of diatoms—is a promising modifier of PLA. One characteristic feature of diatoms is their highly ornamented siliceous shells, called frustules, which are known to have excellent resistance to cracking [3,4]. Recent research results clearly show that diatoms may also impart useful functional properties to diatom-reinforced composite materials [5,6,7].

Composites of PLA modified by DE have been recently studied by Dobrosielska et al. [6]. They demonstrated that addition of diatomaceous earth results in an increase in the tensile strength. Highest mechanical strength has been observed for the composites with 1 wt% of reinforcement, which transforms to 5.5 vol%. Additionally, intact diatoms frustules, obtained by aqua-agriculture, have been used as PLA reinforcement by Li et al. [8]. They reported penetration of the siliceous frustules by polymeric matrix. The results obtained by X-ray diffraction and differential scanning calorimetry analysis revealed that this type of reinforcement acts as a nucleating agent increasing crystallinity. Tensile tests showed enhanced strength and ductility of PLA reinforced by diatom frustules [8].

Generally, the effect of reinforcements depends on their adhesion to the matrix. Aguero et al. [9] investigated several coupling agents such as (3-glycidyloxypropyl) trimethoxysilane, epoxy styrene-acrylic oligomer and maleinized linseed oil [9]. Additions of these compatibilizers result in improvement of the elastic modulus and ductile properties of PLA-DE composites [9]. Gonzalez et al. [10] showed an increase in the ductility of PLA-DE composites by the addition of MLO. PLA-DE composites can be used as filaments in 3D printing [11].

According to Singh et al. [12], screw extrusion technology allows to obtain materials—filaments—for 3D printing. Such approach gives possibility to use variable composition, size and type of reinforcements as well as matrix. Three-dimensional printed materials, especially composite structures, can be applied in various fields such as: biomedical, aerospace, military, automobile [12]. 

In this paper, the effect of silica diatomaceous earth reinforcement on microstructure, melting point, crystallization, thermal degradation and mechanical properties of PLA and DE composites have been investigated. Various kinds of commercial PLA have been used to fabricate composites for eco-friendly applications as of biodegradable plastic products.

## 2. Materials and Methods

### 2.1. Materials

Ingeo^TM^ (NatureWorks, Plymouth, MN, USA) Biopolymers, types of PLA-based materials: 2003D, 3001D, 3251D and 4043D (Nature Works) with a melt flow index (MFI), based on technical data sheets, equal to: 6, 22, 80 and 6, respectively, were used in this study. These Ingeo^TM^ Biolpolymers differ also in suggested processing methods and applications. PLA 2003D can be easily processed via conventional extrusion, whereas 3001D and 3251D are designed for injection molding. Overall, 4043D is well-suited for 3D printing using many different types of printers and for a broad range of printing applications.

Diatomaceous earth (DE, Diatomite, Perma-Guard) with unicellular microscopic organisms (*Aulacoseira* sp.) was used as filler. The single frustule is characterized by a regular, cylindrical shape with numerous openings on the surface. The PLAs and DE were dried at 40 °C under vacuum prior to undertaking fabrication steps.

### 2.2. Fabrication of Composites

The mixtures of PLA and diatomaceous earth (DE) reinforcement (5%, 10% and 15% weight) have been repeatedly pressed using the hydraulic press Fontijne Presses LabEcon300 (Delft, The Netherlands). Each composite has been pressed at least 10 times at a temperature of 190 °C. Afterwards, prepared samples have been manually curated in form of flakes. Reference samples made of PLA only were prepared analogously. 

The flakes were used to manufacture the filaments using the laboratory twin-screw extruder HAAKE MiniLab (ThermoFisher Scientific, Waltham, MA, USA). The process has been conducted at a constant temperature of 175 °C and 50 rpm.

The specific sample information is listed in Table 1.

### 2.3. Experimental Techniques

Observations of the morphology of the DE and its dispersion within the PLA composites were carried out using an ultra-high-resolution analytical dual-beam FIB-SEM tool (Scios2, DualBeam, ThermoFisher, Scientific, Walham, MA, USA) under acceleration voltage of 2 kV. Observations were carried out at magnifications in the range of 500–5000×. For imaging at the highest resolution, samples were coated with a conductive (ca. 7 nm) layer of Cu/Ni using Precision Etching Coating System Model 682 (Gatan, Pleasanton, CA, USA). Observation was carried out of filaments fractured after cooling to the temperature of liquid nitrogen.

The density of the samples has been determined by the Archimedes method using Mettler Toledo XS204 (Columbus, OH, USA) analytical balance equipped with density kit. The density of the PLA samples and their composites was tested with the aid of water (MiliQ water). Average values of density were determined for 10 readings. Theoretical density was calculated assuming the additivity of PLA density and DE true density ρ_DE_ = 2.227 g·cm^−3^ (see Supplementary Information (Tables S1 and S2)) as a weighted sum of both ingredients.

The melting and crystallization behavior of the PLA composites was studied under nitrogen atmosphere (purity 99.999) by differential scanning calorimetry using Q2000 DSC (TA Instruments, New Castle, DE, USA). Each sample of a mass of 7.0 ± 0.1 mg was placed in an aluminum crucible (T_zero_) and held at a temperature of 20 °C for 5 min. Following that, the sample was heated with a heating rate of 10 K·min^−1^ to 200 °C (ca. 30 °C above the PLA matrix melting point of 170 °C) which represents the first heating run. The sample was held 5 min at 200 °C and quenched. After quenching, the sample was held isothermally at 20 °C for 5 min. Subsequently, the sample was heated with a heating rate of 10 K·min^−1^ to 100 °C (second heating run). The cold crystallization enthalpy (ΔH_c_), maximum temperature pick of cold crystallization (T_c_), melting enthalpy (ΔH_m_) and the maximum temperature peak of melting (T_m_) were obtained from the second first heating, the midpoint of the glass transition temperature (T_g_) and change in the heat capacity (ΔC_p_) were obtained from the second heating run. The degree of crystallinity (X_c_) was estimated using the following equation:

(1) $$X_{c}=\frac{\Delta H_{m}-\Delta H_{c}}{\Delta H_{m100\%}\cdot \omega }\cdot 100\%,$$

with ΔH_m__100%_ = 93.7 J·g^−1^ presented by Quero et al. [13]. Melting enthalpy was corrected for filler content (ω).

Static tensile test was carried out according to ISO 527-2:2012 using a Zwick/Roell Z005 (Ulm, Germany) electromechanical testing machine equipped with a load cell with a range of ±1 kN. The test was controlled by a constant displacement of the grips, equal to 15 mm·min^−1^. The initial separation between the grips of the testing machine was 90 mm. A long-range extensometer was used of 40 mm. Average values of parameters were determined for at least five tests. Samples for the static tensile tests had a round beam shape with diameter ca. 2 mm, total length of 90 mm, whereas the measuring length of the extensometer was 50 mm. 

## 3. Results and Discussion

### 3.1. Morphology Analysis and Density Measurements

**Figure 1:**
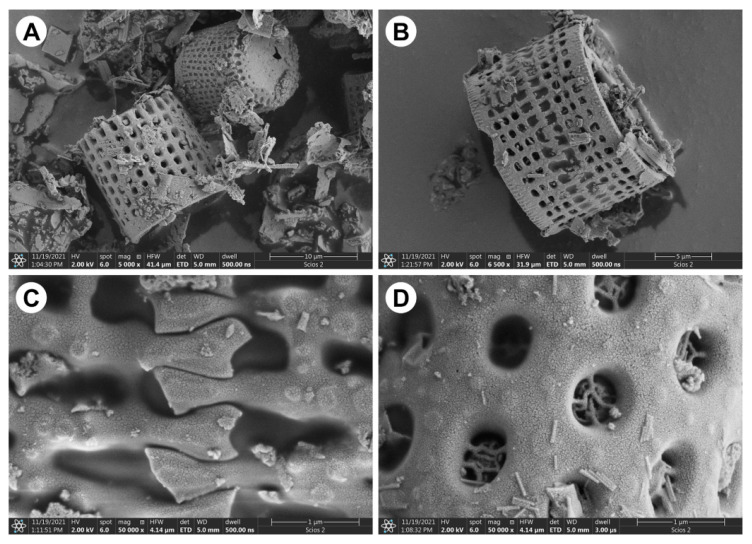
SEM images of the reinforcement—diatomaceous earth: (**A**,**B**) frustules of *Aulacoseira* sp., (**C**) magnification of the zip between frustules, (**D**) magnification of the openings and structures inside the openings.

SEM images of the reinforcement are shown in Figure 1. Figure 1A depicts particles constituting DE used in this study. Figure 1B reveals the morphology of a single diatom frustule characterized by a cylindrical shape with regular openings. The main species within the diatomaceous earth used in the current investigations is *Aulacoseira* sp. This species, found currently in seawaters, feature a zip lock between shells (Figure 1C) and delicate struts reinforcing openings in their frustules (Figure 1D). This locking and struts impart to the frustules’ high strength, taking advantage of which was one of the aims of the present study.

**Figure 2:**
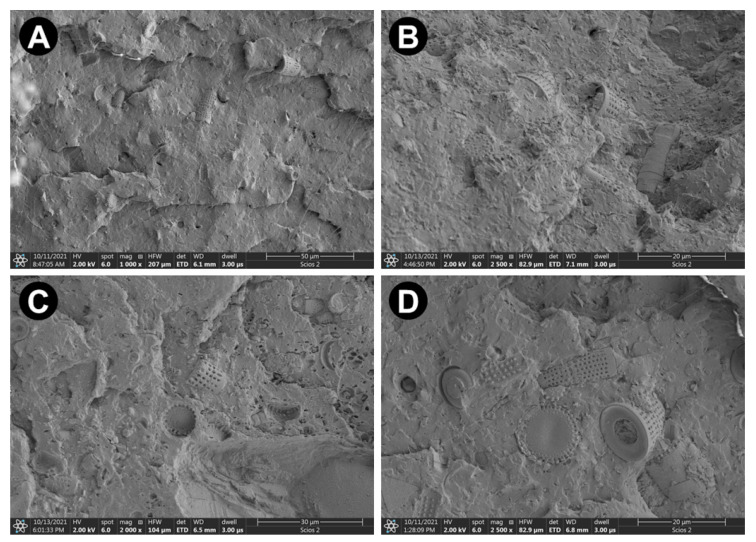
SEM images of the cross-sections of PLA-DE composites showing the uniform distribution of the reinforcement within the polymer matrix (**A**) 2003-5, (**B**) 3001-15, (**C**) 3251-10, (**D**) 4043-10.

Representative images of the composites’ structure prove fairly uniform distribution of the DE within the polymeric matrix (Figure 2 and Figure S2 in the Supplementary Information). Additionally, penetration of the diatoms’ inner cavities by PLA is evidenced. 

**Figure 3:**
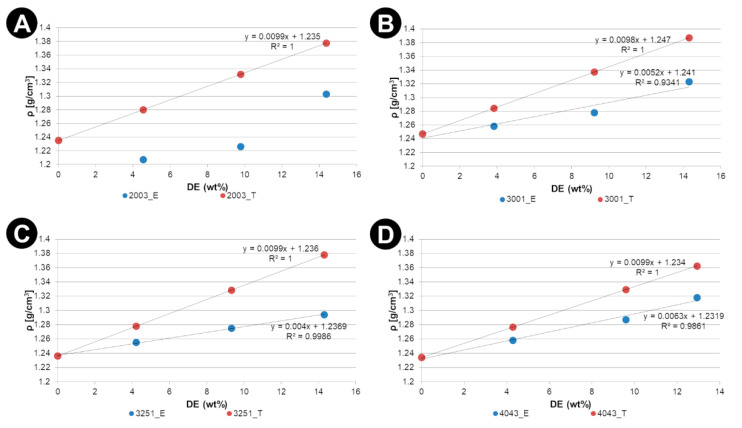
The average density of the manufactured samples against DE weight content in composites with (**A**) 2003D; (**B**) 3001D; (**C**) 3251D and (**D**) 4043D PLA matrices: *E—experimental*, *T—theoretical*.

Results of the measurement of the density of manufactured composites are presented in Figure 3. The results obtained in the measurements of density clearly indicate a considerable porosity, which increases linearly with the content of DE. 

**Figure 4:**
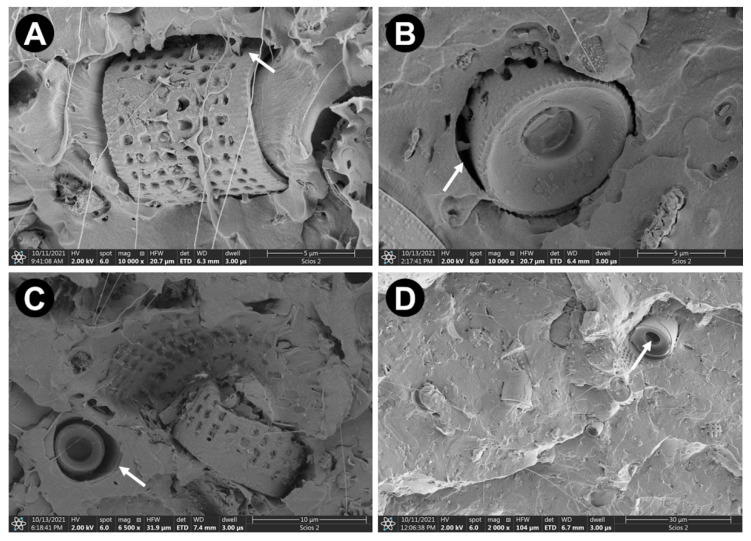
SEM images of the voids (*white arrows*) in manufactured composites: (**A**) 2003-10, (**B**) 3001-5, (**C**) 3251-15, (**D**) 4043-5.

Images in Figure 4 reveal two major forms of the pores/voids: (a) delamination between frustules and PLA matrix, (b) unimpregnated cavities of the frustules. 

### 3.2. Effects of Diatomaceous Earth on the Crystallization of PLA

**Figure 5:**
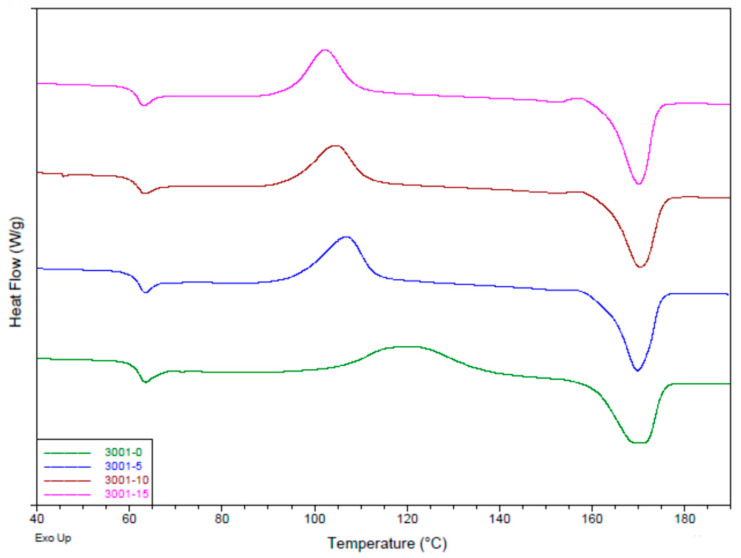
DSC scanning of PLA3001 and 3001 enriched with DE.

The effect of DE reinforcement on properties of PLA matrices is demonstrated by the results of DSC test. DSC first heating curves of the composite with the PLA3001D matrix are shown in Figure 5. Experimental glass transition (T_g_), melting temperature (T_m_), heat capacity and degree of crystallinity (X_c_) are given in Table 2. The DSC first heating curves of other used PLA matrices are presented in the Supplementary Information (see Figure S1).

It can be concluded from DSC tests that addition of DE increased the enthalpy of PLA crystallization phenomena taking place at lower temperatures (*ca*. 107–102 °C), proving the nucleating properties of DE for PLA.

It can be concluded from DSC tests that addition of DE promoted the crystallization phenomena taking place at lower temperatures (*ca*. 107–102 °C), making DE a nucleating agent. Similar phenomenon was observed by Li et al. [6]. This in turn impacts formation of α′ crystals, which are favored at temperatures below 110 °C [11]. The disordered α′ phase is reported to shift to stable form of α crystals at higher temperatures [12]. As a consequence of its looser chain packing and disordered structure, the α′ crystal leads to a lower modulus and barrier properties and to higher elongation at break compared to α crystal [13]. 

A small exothermic peak can be noticed just before the melting peak at samples 3001-5, 3001-10 and 3001-15. This peak was increasingly visible with the growth of the amount of DE in the material. The most probably it occurred due to the transformation of disordered α′ crystals to the ordered α–form [13]. The exothermic peak before melting was visible also in the case of 3251 samples with the addition of DE, whereas the crystallization peak temperature was found to be below 120 °C.

Degree of crystallinity (X_c_) calculated from DSC curves increases with the DE content and ranges from 2.28 to 8.41% and from 5.09 to 8.01% for 2003 and 4043, respectively. The observed effect might be a result of the limited molecular mobility, the 2003 and 4043 melting flow index (MFI) is reported by NatureWorks company to be the same, having a value of 6. Low MFI suggests significant resistance, whereas the polymer chain changes its conformation and retards kinetics of the matrix crystallization. At the same time, samples 3001 and 3251 are characterized by an MFI of 22 and 80 (at 210 °C), representatively. Macromolecules can move easier; therefore, the kinetics of crystallization are expected to be higher. 

The results obtained indicate also that the addition of DE has no significant impact on the melting point. Significant differences of the melting temperatures are ascribed to the additives of commercial PLA materials. Similarly, no effect on glass transition temperature was found.

### 3.3. Effects of Diatomaceous Earth Reinforcement on the Mechanical Properties of PLA

**Figure 6:**
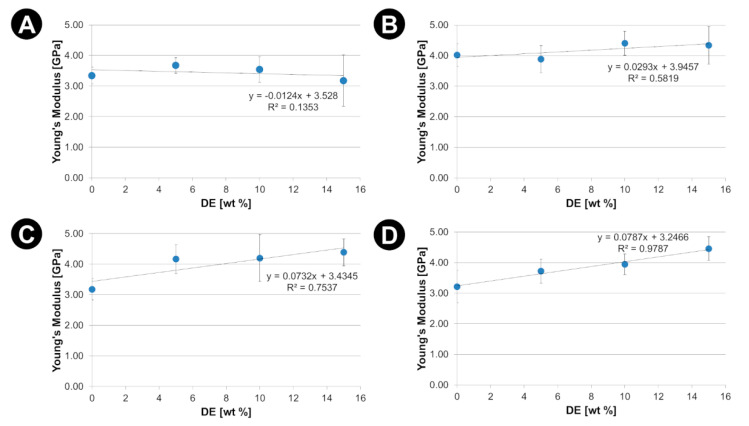
Young’s modulus (E) against the DE content for PLA-DE composites with (**A**) 2003D; (**B**) 3001D; (**C**) 3251D and (**D**) 4043D PLA as a matrix.

**Figure 7:**
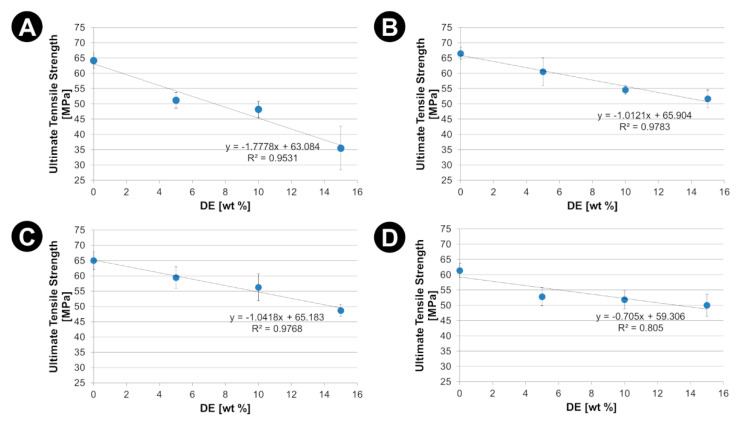
Ultimate tensile strength (R_m_) against the DE content for PLA-DE composites with (**A**) 2003D; (**B**) 3001D; (**C**) 3251D and (**D**) 4043D PLA as a matrix.

**Figure 8:**
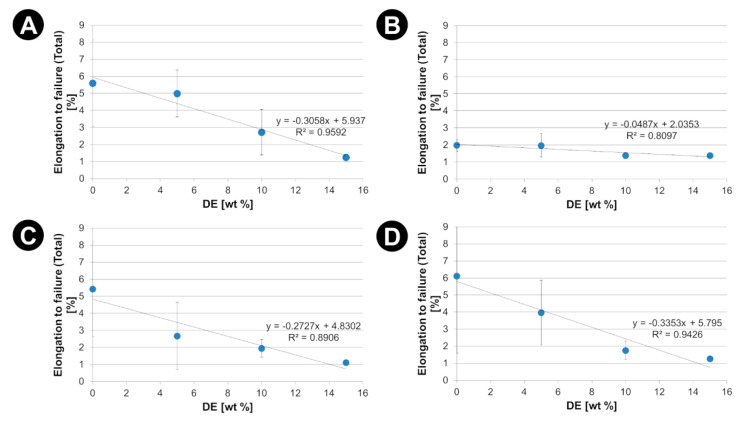
Elongation to failure (ε_f_) against the DE content for PLA-DE composites with (**A**) 2003D; (**B**) 3001D; (**C**) 3251D and (**D**) 4043D PLA as a matrix.

The effects of the diatomaceous earth (DE) as mechanical reinforcement are presented in Figure 6, Figure 7 and Figure 8. The corresponding values of Young’s modulus (E), ultimate tensile strength (R_m_) and elongation to failure (ε_f_) together with the values of the degree of crystallinity have been listed in the Supplementary Information (Table S3). 

**Figure 9:**
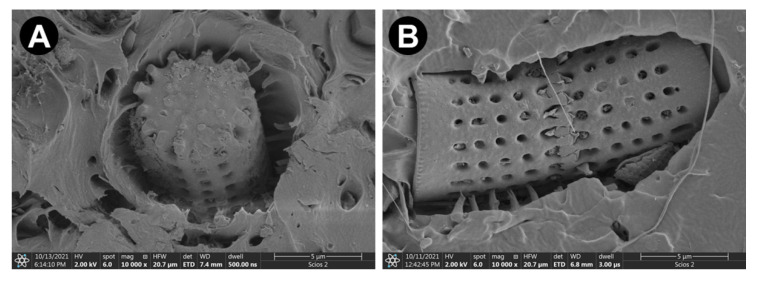
SEM images of the manufactured composites: (**A**) 3251-15, (**B**) 4043-5 showing the created unique structure.

For all PLA used in this study with various MFI ratio, a significant increase in the stiffness of the composites (Figure 6) was observed. This can be explained taking into consideration that DE increase in the degree of crystallinity of the manufactured composites. Such mechanism, also observed by Li et al. [6], is experimentally confirmed by the results presented in Supplementary Information (see Table S3). It is also confirmed by SEM observations of breakthroughs of exemplary composites after applying tensile stress within elastic regime, revealing good adhesion between the matrix and reinforcement (Supplementary Information, see Figure S3). Thus, the increase in the stiffness of the manufactured composites can be strictly connected with the morphology of the diatom’s frustules. The PLA matrix penetrates to a certain depth into the frustules, which adds value to the adhesion, at least in the range of elastic, reversible stresses, see Figure 9. According to Li et al. [6], such interpenetrating structures may enhance the interfacial interaction, resulting in effective stress transfer and energy dissipation between PLA and DE. 

An increase in the crystallinity of the polymer matrix results also in a decrease in its deformation capacity (both elastic and plastic). This effect is confirmed by the significant reduction in the strain to failure (ε_f_) with filler content (Figure 8). This effect seems particularly relevant when the material transitions from the elastic to plastic deformation range. With limited adhesion of the matrix to the DE, where adhesion is mediated by the above-mentioned mechanical interlocking of the PLA matrix in the frustule openings, internal de-cohesion of the composite occurs at the onset of plastic deformation.

## 4. Conclusions

Biodegradable composites in the form of extruded filaments made of various commercial PLA matrices and diatomaceous earth (DE) have been manufactured in a multi-step fabrication process. 

The microstructures of the composites revealed uniform dispersion of the reinforcement in the range of 5–15 weight%. During the process of filaments fabrication PLA matrix partially infiltrated into diatom shells. Such partial infiltration results in increased bonding of the matrix to DE frustules by mechanical locking. On the other hand, cavities of frustule which were not fully filled out bring about frustule-originated “caged” porosity, which reduced the specific weight of the composites without reducing their strength. The comparison of true and theoretical density indicates that the porosity is increasing linearly with the content of DE from 1.5 to 8.6%. However, the increase in the porosity does not reduce elastic modulus, which is either maintained or increases with increasing porosity.

Static tensile tests revealed a complex effect of the DE additives on the mechanical properties of composites. Firstly, withing elastic range of strains a noticeable increase in the stiffness of the composites with the addition of DE was observed, which can be attributed to the specific features of diatoms shells. Diatoms shell have a higher stiffness than PLA matrix as their made of bio-SiO_2_ and have shape rendering high resistance to compression. On the other hand, a decrease in ultimate tensile strength and elongation to failure was observed. This is related to the composite failure mechanism that occurs once the elastic limit is exceeded. In the plastic deformation case, diatoms can be treated as stress concentrators initiating of progressive destruction of composites. In the current study adhesion of diatoms shells to PLA matrix was primarily due to mechanical locking. We believe that further improving the adhesion of PLA to DEs could increase the ultimate tensile strength of the composites.

DE addition also changes the mechanical properties of the PLA matrix (increase in stiffness, limitation of ultimate tensile strength and elongation to failure) due to the effect of an increased degree of crystallinity. DSC results showed that the DE filler acts as a nucleating agent increasing the degree of crystallinity of the PLA matrices. The crystalline phase increases the stiffness of the matrix but at the same time limits its abilities in large (plastic) deformation, which consequently causes a limitation of ultimate tensile strength.

The conducted investigations showed that biogenic filler in the form of diatomaceous earth can be used as an environmentally friendly additive in biodegradable PLA matrices. Possible applications of these bio-composites are filaments for 3D printing. It should be noted in this context that PLA is one of the most popular filaments used for Fused Deposition Modelling (FDM) printing. The performance of the fabricated filaments in 3D printing is the subject of ongoing investigations.

## Figures and Tables

**Table 1:** The codes and composition of the prepared composite materials.

Sample	PLA (wt%)	DE (wt%)
2003-0	100	0
2003-5	95	5
2003-10	90	10
2003-15	85	15
3001-0	100	0
3001-5	95	5
3001-10	90	10
3001-15	85	15
3251-0	100	0
3251-5	95	5
3251-10	90	10
3251-15	85	15
4043-0	100	0
4043-5	95	5
4043-10	90	10
4043-15	85	15

**Table 2:** DSC results for the PLA and the PLA–DE composites.

Sample	T_g_ [°C]	ΔC_p_ [J·(g·°C)^−1^]	T_c_ [°C]	ΔH_c_ [J·g^−1^]	T_m_ [°C]	ΔH_m_ [J·g^−1^]	X_c_ [%]
2003-0	60.7	0.755	119.6	19.26	151.6	21.38	2.28
2003-5	61.1	0.734	125.1	11.35	151.9	15.76	4.97
2003-10	61.1	0.708	121.1	7.16	151.1	13.29	7.30
2003-15	60.9	0.691	117.4	11.06	151.3	17.76	8.41
3001-0	61.9	0.710	121.0	33.34	169.2	41.44	8.69
3001-5	62.3	0.621	107.1	33.09	169.7	41.25	9.10
3001-10	62.2	0.599	104.6	29.69	170.4	37.69	9.45
3001-15	62.1	0.618	102.1	28.56	170.1	37.22	10.84
3251-0	61.7	0.719	107.3	36.46	171.7	44.58	8.75
3251-5	61.6	0.572	103.0	31.77	170.5	41.44	10.88
3251-10	61.4	0.683	102.1	33.11	170.5	45.12	14.28
3251-15	61.7	0.549	101.5	30.20	170.1	40.66	13.16
4043-0	61.5	0.759	122.3	11.90	151.8	16.63	5.09
4043-5	61.3	0.688	126.0	13.56	152.3	18.28	5.30
4043-10	61.4	0.685	123.9	13.50	152.7	18.72	6.21
4043-15	60.9	0.634	122.7	7.99	152.8	14.48	8.01

where (see Section 2.3 Experimental techniques for details): T_g_ the glass transition temperature; ΔC_p_ change in the heat capacity; T_c_ maximum temperature pick of cold crystallization; ΔH_c_ the cold crystallization enthalpy; T_m_ the maximum temperature peak of melting; ΔH_m_ melting enthalpy; X_c_ the degree of crystallinity.

## Data Availability

All data generated or analyzed during this study are included in this published article (and its Supplementary Information Files).

## Supplementary Materials

The following supporting information can be downloaded at: https://www.mdpi.com/article/10.3390/ma15186210/s1, Experimental section: description of true density; Figure S1. DSC scans of the 1st heating of manufactured composites PLA-DE with (A) Ingeo™ Biopolymer 2003D, (B) Ingeo™ Biopolymer 3251D and (C) Ingeo™ Biopolymer 4043D PLA as a matrix; Figure S2. SEM images of breakthroughs of composites with (A–C) PLA2003D, (D–F) PLA3001D, (G–I) PLA3251D, (J–L) PLA4043D matrix and (A,D,G,J) 5 wt% DE, (B,E,H,K) 10 wt% DE and (C,F,I,L) 15 wt% DE reinforcement; Figure S3. SEM images of breakthroughs of composites (A,B) PLA4043D–5 wt% and (C,D) PLA4043D–15 wt% after tensile tests in elastic range; Table S1. The results of the true density; Table S2. The theoretical and experimental composition as well as density of PLA and PLA–DE composites; Table S3. Tensile results and degree of the crystallinity for the PLAs and the PLA/DE composites.
