# Supplementary material for: Poly(lactic acid) Matrix Reinforced with Diatomaceous Earth
Source: Materials (Basel). 2022 Sep 7;15(18):6210. doi: 10.3390/ma15186210 (PMC9501419; doi:10.3390/ma15186210)
Supplement: Supplementary file 1 [file materials-15-06210-s001.zip › materials-1853978-supplementary.pdf]

Supporting Information

PLA matrix composites reinforced with diatomaceous earth

Izabela Zgłobicka, Magdalena Joka, Rafał Molak, Michał Kawalec, Adrian Dubicki, Jakub Wróblewski, Kamil Dydek, Anna Boczkowska, Krzysztof J. Kurzydlowski*

Experimental section

True density

The true density has been measured using a micrometritics AccuPhyc II 1340 Gas Pycnometer (accreditation No. AB 1503: from 0.7 cm^3 to 7 cm^3) using a Boyle's law as below (1):

$$V_S = V_C - \frac{V_E}{P1P2-1} \quad (1)$$

where V_S is the volume of the sample, V_C is the volume of the sample's cell, and V_E is the volume of the expansion cell. After that, the true density of the sample was calculated by using the formula (2):

$$\rho_t = \frac{m}{V_S} \quad (2)$$

where ρ_t is the true density and m is the mass of the sample. As for the true density, it measures just the volume of the solid materials and excludes the volume of all open pores. The experiments have been conducted in the atmosphere of the inert gas - helium (purity ≥ 99.999%). The analysis was carried out with the usage of the FoamPyc V1.06 software.

The obtained results are presented in Table S1. The measurement uncertainty was determined in accordance with the document EA-4/02 M:2013. The given uncertain values represent the expanded uncertainties with a coverage probability of approx. 95% and a coverage factor k = 2. The expanded uncertainty was determined in accordance with the procedure P5.12 and with the document EA-4/16.

Figures

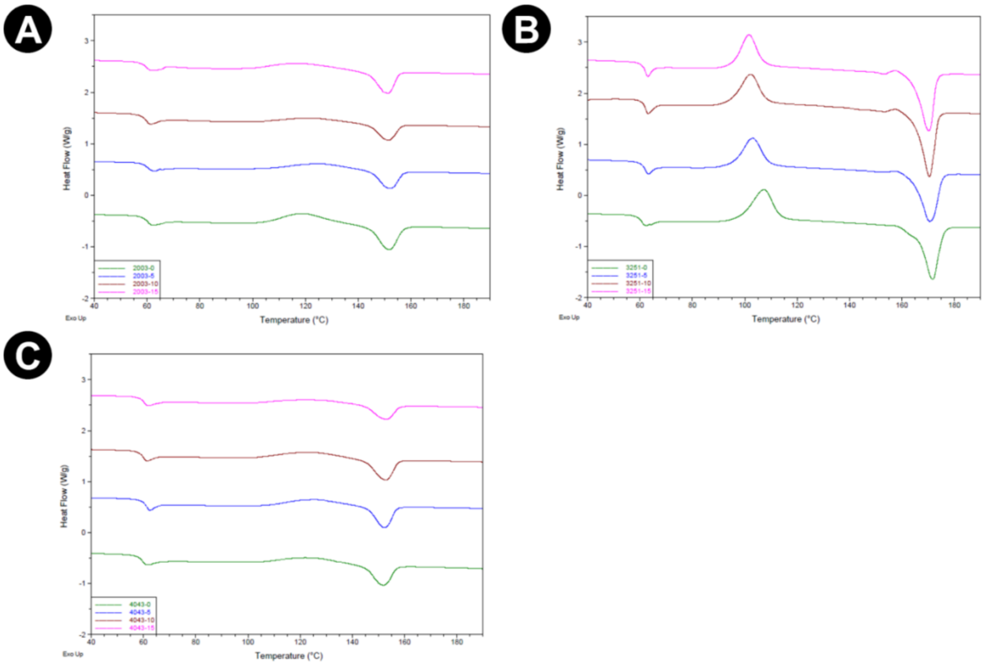

Figure S1. DSC scans of the 1st heating of manufactured composites PLA-DE with (A) Ingeo™ Biopolymer 2003D, (B) Ingeo™ Biopolymer 3251D and (C) Ingeo™ Biopolymer 4043D PLA as a matrix

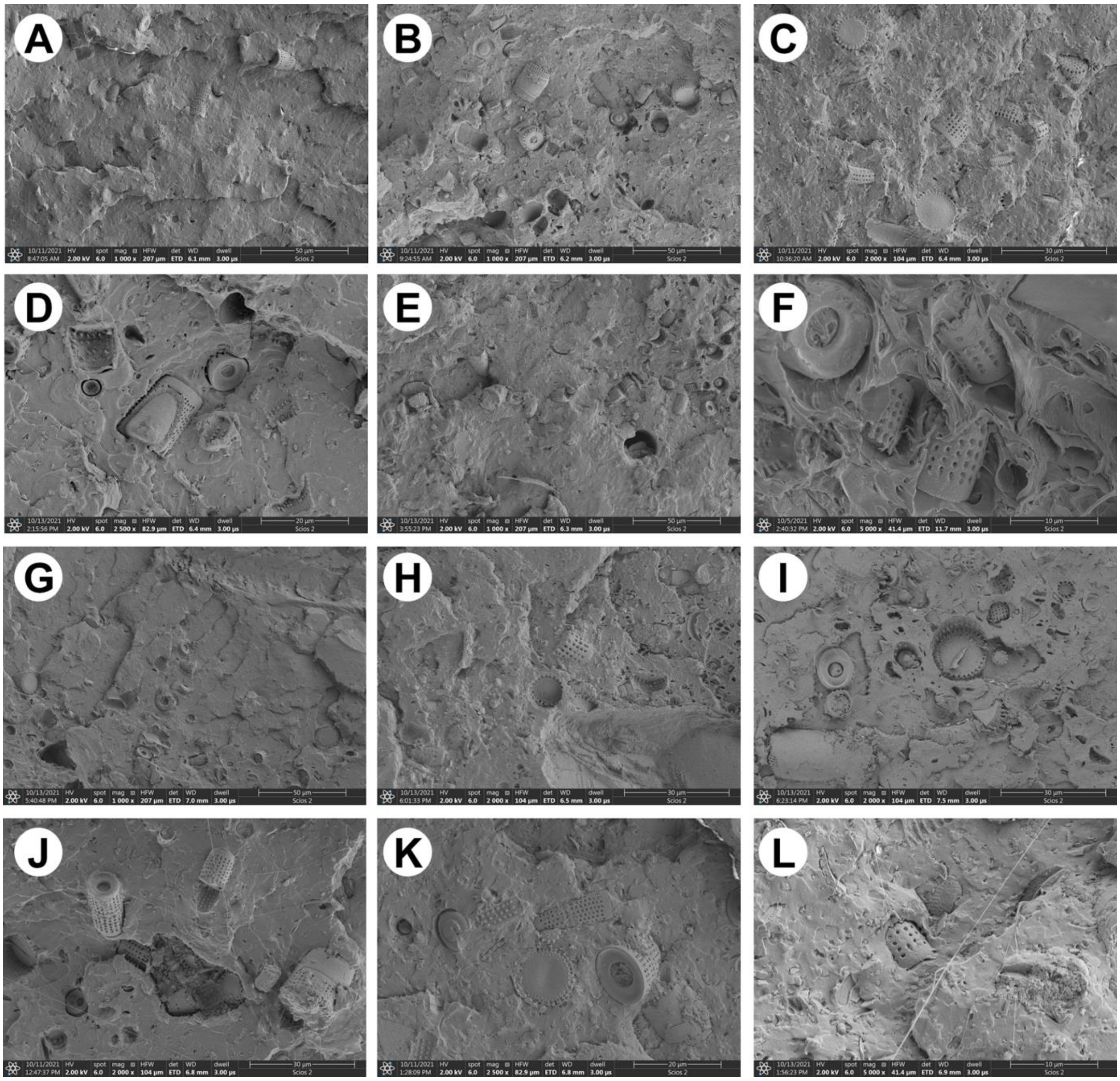

Figure S2. SEM images of breakthroughs of composites with (A-C) PLA2003D, (D-F) PLA3001D, (G-I) PLA3251D, (J-L) PLA4043D matrix and (A,D,G,J) 5wt% DE, (B,E,H,K) 10wt% DE and (C,F,I,L) 15wt%DE reinforcement

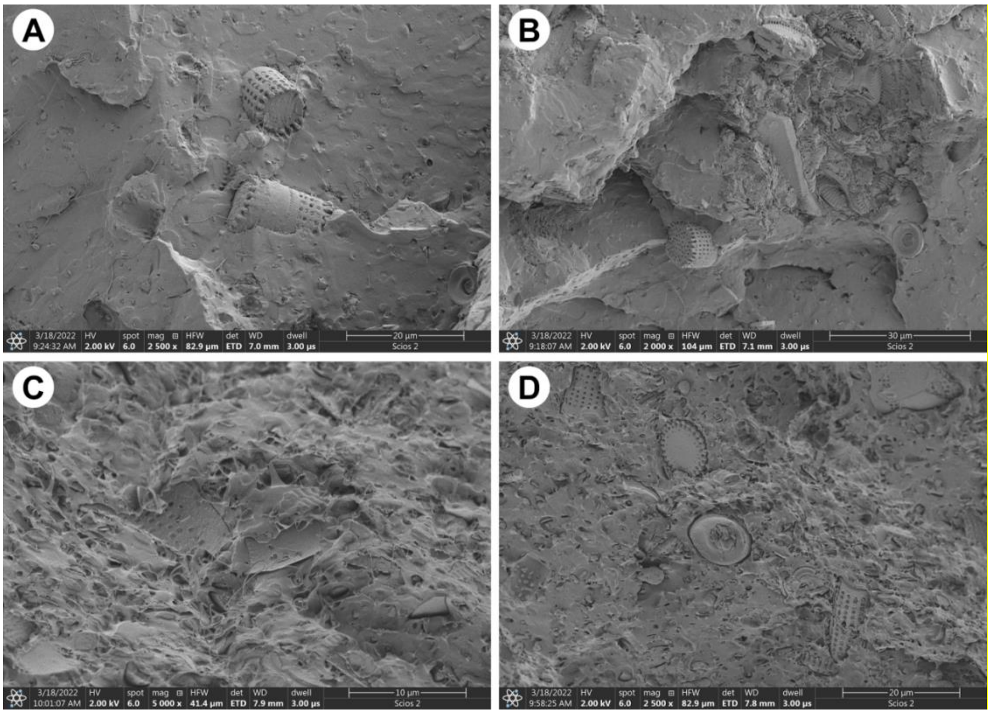

Figure S3. SEM images of breakthroughs of composites (A-B) PLA4043D–5wt% and (C-D) PLA4043D–15wt% after tensile tests in elastic range.

Tables

Table S1. The results of the true density

Sample	ϱ_s [g·cm^{-3}]	SD(ϱ_s) [g·cm^{-3}]	U_c(ϱ_s) [g·cm^{-3}]	V_s ± SD_v [cm^3]	T + SD_{temp} [°C]
diatomaceous earth	2.227	0.005	0.012	(0.2188±0.0005)*	29.3±0.1

Parameters:

- ϱ_s [g·cm^{-3}] – true density
- SD(ϱs) [g·cm^{-3}] – standard deviation of density
- T [°C] – average temperature in the measuring chamber
- SD_{temp} [°C] – standard deviation of temperature in the measuring chamber
- Uc(ϱs) [g·cm^{-3}] – total expanded uncertainty of density
- V_s [cm^3] – true volume of the sample
- SD_V [cm^3] – standard deviation of the true volume of the sample

*result outside the scope of accreditation No.AB 1503

Table S2. The theoretical and experimental composition as well as density of PLA and PLA–DE composites

Sample	Theoretical composition		Composition determined by TGA		Determined ϱ [g·cm^{-3}] Archimedes method	Theoretical ϱ_{th} [g·cm^{-3}]	$\Delta\varrho$ ϱ_{th} - ϱ [g·cm^{-3}]	$\Delta\varrho$/[DE]
	PLA (wt%)	DE (wt%)	PLA (wt%)	DE (wt%)				
2003-0	100	0	100	0.00	1.235±0.004			
2003-5	95	5	95.46	4.54	1.207±0.019	1.285	0.078	0.017
2003-10	90	10	90.23	9.77	1.226±0.016	1.334	0.108	0.011
2003-15	85	15	85.63	14.37	1.303±0.003	1.384	0.081	0.006
3001-0	100	0	100	0.00	1.247±0.005			
3001-5	95	5	96.17	3.83	1.258±0.009	1.296	0.038	0.010
3001-10	90	10	90.78	9.22	1.278±0.010	1.345	0.067	0.007
3001-15	85	15	85.70	14.3	1.323±0.009	1.394	0.071	0.005
3251-0	100	0	100	0.00	1.236±0.005			
3251-5	95	5	95.79	4.21	1.255±0.008	1.286	0.031	0.007
3251-10	90	10	90.67	9.33	1.275±0.017	1.335	0.060	0.006
3251-15	85	15	85.69	14.31	1.294±0.016	1.385	0.091	0.006
4043-0	100	0	100	0.00	1.234±0.004			
4043-5	95	5	95.72	4.28	1.258±0.007	1.284	0.036	0.008
4043-10	90	10	90.41	9.59	1.287±0.002	1.333	0.046	0.005
4043-15	85	15	87.07	12.93	1.318±0.005	1.383	0.065	0.005

Table S3. Tensile results and degree of the crystallinity for the PLAs and the PLA/DE composites

Sample	E [GPa]	R_m [MPa]	ε_f [%]	X_c [%]
2003-0	3.34±0.28	64.17±2.54	5.59±2.53	2.28
2003-5	3.67±0.26	51.14±2.60	5.00±1.38	4.97
2003-10	3.54±0.42	48.16±2.77	2.73±1.33	7.30
2003-15	3.18±0.84	35.53±7.19	1.25±0.18	8.41
3001-0	4.02±0.38	66.51±1.98	1.98±0.34	8.69
3001-5	3.89±0.45	60.55±4.60	1.96±0.68	9.10
3001-10	4.41±0.39	54.54±1.43	1.37±0.11	9.45
3001-15	4.34±0.62	51.65±2.88	1.37±0.17	10.84
3251-0	3.18±0.35	65.02±2.90	5.41±2.78	8.75
3251-5	4.16±0.47	59.46±3.51	2.67±1.97	10.88
3251-10	4.20±0.77	56.26±4.40	1.95±0.51	14.28
3251-15	4.39±0.44	48.73±1.94	1.11±0.16	13.16
4043-0	3.22±0.53	61.42±2.38	6.12±4.53	5.09
4043-5	3.73±0.40	52.82±2.96	3.97±1.90	5.30
4043-10	3.95±0.34	51.83±2.96	1.76±0.55	6.21
4043-15	4.46±0.39	50.00±3.65	1.27±0.14	8.01
